# 1-Formyl-*c*-3,*t*-3-dimethyl-*r*-2,*c*-6-di­phenyl­piperidin-4-one

**DOI:** 10.1107/S1600536812007015

**Published:** 2012-02-29

**Authors:** K. Ravichandran, P. Sakthivel, P. Ramesh, S. Ponnuswamy, M. N. Ponnuswamy

**Affiliations:** a Centre of Advanced Study in Crystallography and Biophysics, University of Madras, Guindy Campus, Chennai 600 025, India; b Department of Chemistry, Government Arts College (Autonomous), Coimbatore 641 018, India

## Abstract

In the title compound, C_20_H_21_NO_2_, the piperidine ring adopts a distorted boat conformation. The phenyl rings substituted at the 2- and 6-positions of the piperidine ring subtend angles of 86.0 (1) and 67.3 (1)° with the mean plane of the piperidine ring (all six non-H atoms). The crystal packing features C—H⋯O inter­actions.

## Related literature

For the biological activity of piperidine derivatives, see: Aridoss *et al.* (2009 ▶); Nalanishi *et al.* (1974 ▶); Michael (2001 ▶); Pinder (1992 ▶); Rubiralta *et al.* (1991 ▶). For puckering parameters, see: Cremer & Pople (1975 ▶). For asymmetry parameters, see: Nardelli (1983 ▶). For hydrogen-bond motifs, see: Bernstein *et al.*(1995 ▶).
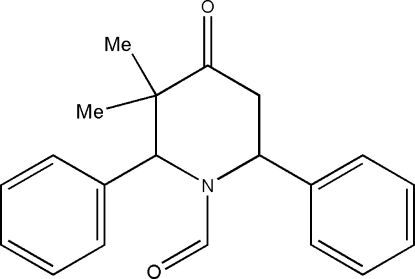


## Experimental

### 

#### Crystal data

C_20_H_21_NO_2_
*M* *_r_* = 307.38
Monoclinic, 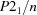

*a* = 10.6604 (6) Å
*b* = 15.7278 (7) Å
*c* = 10.8066 (5) Å
β = 110.120 (2)°
*V* = 1701.31 (15) Å^3^
*Z* = 4
Mo *K*α radiation
μ = 0.08 mm^−1^
*T* = 293 K
0.20 × 0.20 × 0.20 mm

#### Data collection

Bruker SMART APEX CCD detector diffractometer
Absorption correction: multi-scan (*SADABS*; Bruker, 1998 ▶) *T*_min_ = 0.985, *T*_max_ = 0.985
16240 measured reflections
4162 independent reflections
2769 reflections with *I* > 2σ(*I*)
*R*_int_ = 0.021

#### Refinement

*R*[*F* ^2^ > 2σ(*F* ^2^)] = 0.042
*wR*(*F* ^2^) = 0.125
*S* = 1.04
4162 reflections
209 parameters
H-atom parameters constrained
Δρ_max_ = 0.19 e Å^−3^
Δρ_min_ = −0.13 e Å^−3^

### 

Data collection: *SMART* (Bruker, 1998 ▶); cell refinement: *SAINT-Plus* (Bruker, 1998 ▶); data reduction: *SAINT-Plus*; program(s) used to solve structure: *SHELXS97* (Sheldrick, 2008 ▶); program(s) used to refine structure: *SHELXL97* (Sheldrick, 2008 ▶); molecular graphics: *ORTEP-3* (Farrugia, 1997 ▶); software used to prepare material for publication: *SHELXL97* and *PLATON* (Spek, 2009 ▶).

## Supplementary Material

Crystal structure: contains datablock(s) global, I. DOI: 10.1107/S1600536812007015/bt5783sup1.cif

Structure factors: contains datablock(s) I. DOI: 10.1107/S1600536812007015/bt5783Isup2.hkl

Supplementary material file. DOI: 10.1107/S1600536812007015/bt5783Isup3.cml

Additional supplementary materials:  crystallographic information; 3D view; checkCIF report

## Figures and Tables

**Table 1:** Hydrogen-bond geometry (Å, °)

*D*—H⋯*A*	*D*—H	H⋯*A*	*D*⋯*A*	*D*—H⋯*A*
C6—H6⋯O1^i^	0.98	2.59	3.2951 (17)	129

Symmetry code: (i) 
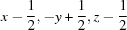
.

**Fig. 1.**
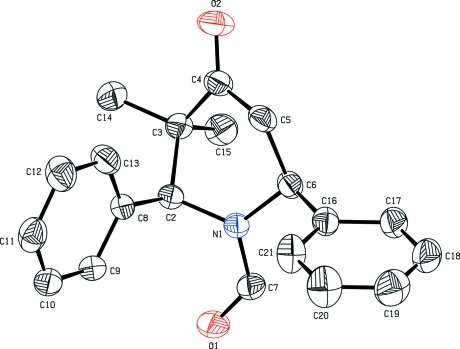
The molecular structure of the title compound, showing the atomic numbering and displacement ellipsoids drawn at 50% probability level.

**Fig. 2.**
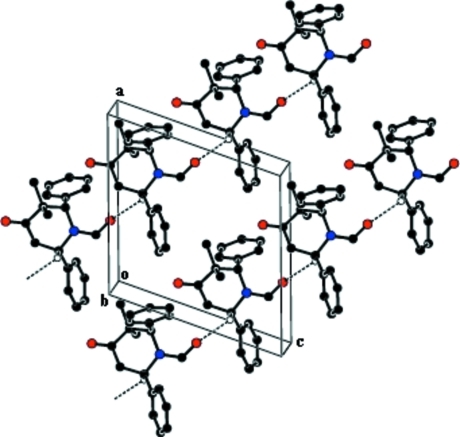
The crystal packing of the molecules. H atoms not involved in hydrogen bonding (dashed lines) have been omitted for clarity.

## supplementary crystallographic information

### Comment

Piperidine derivatives are the valued heterocyclic compounds in the field of medicinal chemistry. The compounds possessing an amide bond linkage have a wide range of biological activities such as antimicrobial, anti-inflammatory, antiviral, antimalarial and general anesthetics (Aridoss *et al.*, 2009). Functionalized piperidines are familiar substructures found in biologically active natural products and synthetic pharmaceuticals (Michael, 2001; Pinder, 1992; Rubiralta *et al.*, 1991). Piperidines have been found to exhibit blood cholesterol-lowering activities (Nalanishi *et al.*, 1974). Against this background and to ascertain the molecular structure and conformation, the X-ray crystal structure determination of the title compound has been carried out.

The *ORTEP* plot of the molecule is shown in Fig. 1. The formyl substituted piperidine derivative crystallizes in monoclinic space groups *P*2_1_/*n*. The piperidine ring adopts distorted boat conformation with the puckering parameters (Cremer & Pople, 1975) and the asymmetry parameters (Nardelli, 1983) are: q_2_=0.648 (2) Å, q_3_ = -0.047 (1) Å, φ_2_ = -98.9 (1)° and Δ_s_(N1 & C4)= 16.8 (1)°. The sum of the bond angles around N1 [359.2°] is in accordance with *sp^2^* hybridization.

The carbonyl group is oriented *syn* to C2 [C2—N1—C7—O1=] -6.5 (2)° and *anti* to C6 [C6—N1—C7—O1=] -176.7 (1)°. The best plane of the piperidine ring and the attached phenyl rings [C8—C13 & C16—C21] are twisted away by 86.0 (1)° & 67.3 (1)°. The two phenyl rings are approximately perpendicular to each other with a dihedral angle of 86.5 (1)°.

The crystal packing reveals that the symmetry related molecules are linked through a network of C—H···O type of intermolecular interactions. The atom C6 at (*x*, *y*, *z*) donates a proton to O1 at (-1/2 + *x*, 1/2 - *y*, -1/2 + *z*) forming a C(5) one dimensional chain running along the *ac* diagonal axis (Bernstein *et al.*, 1995).

### Experimental

To ice-cold acetic anhydride (10 ml), 85% formic acid (5 ml) was added slowly and the resulting acetic acid–formic anhydride was cooled to 5° C and added slowly to a cold solution of piperidine-4-one (5 mmol) in anhydrous benzene (30 ml). The reaction mixture was stirred at room temperature for 5 h and the solution was poured into water (250 ml). The benzene layer was separated out, concentrated and recrystallized from benzene: pet-ether (60–80° C) in the ratio 1:1.

### Refinement

H atoms were positioned geometrically (C–H = 0.93–0.97 Å) and allowed to ride on their parent atoms, with *U*_iso_(H) = 1.5*U*_eq_(C) for methyl H atoms and 1.2*U*_eq_(C) for all other H atoms.

### Crystal data

**Table:**

C_20_H_21_NO_2_	*F*(000) = 656
*M**_r_* = 307.38	*D*_x_ = 1.200 Mg m^−^^3^
Monoclinic, *P*2_1_/*n*	Mo *K*α radiation, λ = 0.71073 Å
Hall symbol: -P 2yn	Cell parameters from 2769 reflections
*a* = 10.6604 (6) Å	θ = 2.3–28.3°
*b* = 15.7278 (7) Å	µ = 0.08 mm^−^^1^
*c* = 10.8066 (5) Å	*T* = 293 K
β = 110.120 (2)°	Block, yellow
*V* = 1701.31 (15) Å^3^	0.20 × 0.20 × 0.20 mm
*Z* = 4	

### Data collection

**Table:**

Bruker SMART APEX CCD detector diffractometer	4162 independent reflections
Radiation source: fine-focus sealed tube	2769 reflections with *I* > 2σ(*I*)
Graphite monochromator	*R*_int_ = 0.021
ω scans	θ_max_ = 28.3°, θ_min_ = 2.3°
Absorption correction: multi-scan (*SADABS*; Bruker, 1998)	*h* = −13→9
*T*_min_ = 0.985, *T*_max_ = 0.985	*k* = −17→20
16240 measured reflections	*l* = −14→14

### Refinement

**Table:**

Refinement on *F*^2^	Secondary atom site location: difference Fourier map
Least-squares matrix: full	Hydrogen site location: inferred from neighbouring sites
*R*[*F*^2^ > 2σ(*F*^2^)] = 0.042	H-atom parameters constrained
*wR*(*F*^2^) = 0.125	*w* = 1/[σ^2^(*F*_o_^2^) + (0.0489*P*)^2^ + 0.2736*P*] where *P* = (*F*_o_^2^ + 2*F*_c_^2^)/3
*S* = 1.04	(Δ/σ)_max_ < 0.001
4162 reflections	Δρ_max_ = 0.19 e Å^−^^3^
209 parameters	Δρ_min_ = −0.13 e Å^−^^3^
0 restraints	Extinction correction: *SHELXL97* (Sheldrick, 2008), Fc^*^=kFc[1+0.001xFc^2^λ^3^/sin(2θ)]^-1/4^
Primary atom site location: structure-invariant direct methods	Extinction coefficient: 0.0063 (15)

### Special details

**Table:**

Geometry. All e.s.d.'s (except the e.s.d. in the dihedral angle between two l.s. planes) are estimated using the full covariance matrix. The cell e.s.d.'s are taken into account individually in the estimation of e.s.d.'s in distances, angles and torsion angles; correlations between e.s.d.'s in cell parameters are only used when they are defined by crystal symmetry. An approximate (isotropic) treatment of cell e.s.d.'s is used for estimating e.s.d.'s involving l.s. planes.
Refinement. Refinement of *F*^2^ against ALL reflections. The weighted *R*-factor *wR* and goodness of fit *S* are based on *F*^2^, conventional *R*-factors *R* are based on *F*, with *F* set to zero for negative *F*^2^. The threshold expression of *F*^2^ > σ(*F*^2^) is used only for calculating *R*-factors(gt) *etc*. and is not relevant to the choice of reflections for refinement. *R*-factors based on *F*^2^ are statistically about twice as large as those based on *F*, and *R*- factors based on ALL data will be even larger.

### Fractional atomic coordinates and isotropic or equivalent isotropic displacement parameters (Å^2^)

**Table:**

	*x*	*y*	*z*	*U*_iso_*/*U*_eq_	
O1	0.33930 (11)	0.19983 (7)	0.96574 (9)	0.0689 (3)	
O2	0.12616 (13)	0.21093 (8)	0.35746 (10)	0.0845 (4)	
N1	0.20536 (10)	0.17426 (7)	0.75311 (10)	0.0487 (3)	
C2	0.31776 (13)	0.17185 (9)	0.70280 (12)	0.0483 (3)	
H2	0.3914	0.2017	0.7688	0.058*	
C3	0.28375 (14)	0.22558 (9)	0.57618 (13)	0.0554 (4)	
C4	0.15392 (15)	0.19561 (10)	0.47344 (13)	0.0597 (4)	
C5	0.05906 (14)	0.14749 (10)	0.52365 (12)	0.0598 (4)	
H5A	0.0781	0.0872	0.5226	0.072*	
H5B	−0.0312	0.1567	0.4633	0.072*	
C6	0.06466 (13)	0.17169 (9)	0.66235 (13)	0.0530 (3)	
H6	0.0275	0.2290	0.6582	0.064*	
C7	0.22970 (15)	0.19192 (9)	0.88088 (13)	0.0563 (4)	
H7	0.1558	0.1988	0.9071	0.068*	
C8	0.36733 (12)	0.08118 (9)	0.69907 (12)	0.0477 (3)	
C9	0.45199 (14)	0.04765 (10)	0.81673 (13)	0.0583 (4)	
H9	0.4758	0.0808	0.8926	0.070*	
C10	0.50188 (17)	−0.03415 (12)	0.82365 (16)	0.0715 (5)	
H10	0.5580	−0.0555	0.9038	0.086*	
C11	0.46891 (17)	−0.08374 (11)	0.71304 (19)	0.0732 (5)	
H11	0.5018	−0.1389	0.7177	0.088*	
C12	0.38724 (18)	−0.05151 (12)	0.59586 (18)	0.0781 (5)	
H12	0.3655	−0.0847	0.5201	0.094*	
C13	0.33629 (16)	0.03008 (11)	0.58839 (15)	0.0683 (4)	
H13	0.2803	0.0508	0.5077	0.082*	
C14	0.40046 (17)	0.22591 (12)	0.52432 (16)	0.0751 (5)	
H14A	0.4797	0.2455	0.5923	0.113*	
H14B	0.3801	0.2631	0.4495	0.113*	
H14C	0.4148	0.1693	0.4986	0.113*	
C15	0.25767 (18)	0.31820 (10)	0.60864 (17)	0.0709 (4)	
H15A	0.1843	0.3194	0.6409	0.106*	
H15B	0.2363	0.3523	0.5304	0.106*	
H15C	0.3362	0.3404	0.6747	0.106*	
C16	−0.01874 (13)	0.11249 (10)	0.71207 (13)	0.0553 (4)	
C17	−0.14145 (15)	0.13908 (12)	0.71422 (15)	0.0681 (4)	
H17	−0.1720	0.1935	0.6854	0.082*	
C18	−0.21912 (18)	0.08524 (16)	0.75901 (18)	0.0855 (6)	
H18	−0.3018	0.1035	0.7599	0.103*	
C19	−0.1747 (2)	0.00528 (16)	0.8021 (2)	0.0905 (6)	
H19	−0.2269	−0.0307	0.8326	0.109*	
C20	−0.0538 (2)	−0.02166 (14)	0.8001 (2)	0.0942 (6)	
H20	−0.0238	−0.0761	0.8291	0.113*	
C21	0.02406 (18)	0.03149 (12)	0.75529 (19)	0.0774 (5)	
H21	0.1063	0.0126	0.7542	0.093*	

### Atomic displacement parameters (Å^2^)

**Table:**

	*U*^11^	*U*^22^	*U*^33^	*U*^12^	*U*^13^	*U*^23^
O1	0.0658 (7)	0.0879 (8)	0.0450 (5)	−0.0068 (6)	0.0089 (5)	−0.0128 (5)
O2	0.0962 (9)	0.1036 (10)	0.0445 (6)	0.0147 (7)	0.0125 (5)	0.0114 (5)
N1	0.0453 (6)	0.0573 (7)	0.0408 (5)	0.0030 (5)	0.0113 (4)	−0.0060 (5)
C2	0.0450 (7)	0.0562 (8)	0.0407 (6)	0.0011 (6)	0.0112 (5)	−0.0026 (5)
C3	0.0598 (8)	0.0586 (9)	0.0485 (7)	0.0094 (7)	0.0194 (6)	0.0058 (6)
C4	0.0674 (9)	0.0618 (9)	0.0452 (7)	0.0186 (7)	0.0135 (6)	0.0028 (6)
C5	0.0524 (8)	0.0704 (10)	0.0453 (7)	0.0085 (7)	0.0024 (6)	−0.0057 (6)
C6	0.0457 (7)	0.0581 (9)	0.0497 (7)	0.0070 (6)	0.0095 (5)	−0.0057 (6)
C7	0.0590 (8)	0.0627 (9)	0.0473 (7)	−0.0003 (7)	0.0182 (6)	−0.0084 (6)
C8	0.0401 (6)	0.0572 (8)	0.0456 (6)	0.0030 (6)	0.0144 (5)	0.0026 (5)
C9	0.0570 (8)	0.0741 (10)	0.0458 (7)	0.0094 (7)	0.0201 (6)	0.0095 (6)
C10	0.0744 (10)	0.0827 (12)	0.0666 (9)	0.0262 (9)	0.0358 (8)	0.0315 (8)
C11	0.0781 (11)	0.0592 (10)	0.0960 (12)	0.0132 (8)	0.0477 (10)	0.0143 (9)
C12	0.0754 (11)	0.0703 (12)	0.0831 (11)	0.0124 (9)	0.0204 (9)	−0.0172 (9)
C13	0.0650 (9)	0.0731 (11)	0.0552 (8)	0.0185 (8)	0.0057 (7)	−0.0085 (7)
C14	0.0763 (11)	0.0899 (13)	0.0677 (9)	0.0150 (9)	0.0357 (8)	0.0199 (8)
C15	0.0842 (11)	0.0568 (10)	0.0740 (10)	0.0053 (8)	0.0302 (9)	0.0061 (7)
C16	0.0469 (7)	0.0628 (9)	0.0522 (7)	−0.0006 (7)	0.0117 (6)	−0.0136 (6)
C17	0.0517 (8)	0.0876 (12)	0.0619 (9)	0.0046 (8)	0.0157 (7)	−0.0091 (8)
C18	0.0546 (10)	0.1252 (18)	0.0783 (11)	−0.0028 (11)	0.0249 (8)	−0.0087 (11)
C19	0.0761 (13)	0.1046 (17)	0.0937 (13)	−0.0247 (12)	0.0330 (10)	−0.0060 (12)
C20	0.0880 (14)	0.0718 (13)	0.1283 (17)	−0.0076 (10)	0.0441 (13)	0.0039 (11)
C21	0.0652 (10)	0.0650 (11)	0.1073 (13)	0.0013 (8)	0.0365 (10)	−0.0046 (9)

### Geometric parameters (Å, º)

**Table:**

O1—C7	1.2183 (17)	C10—H10	0.9300
O2—C4	1.2088 (16)	C11—C12	1.364 (2)
N1—C7	1.3433 (16)	C11—H11	0.9300
N1—C2	1.4768 (16)	C12—C13	1.385 (2)
N1—C6	1.4837 (16)	C12—H12	0.9300
C2—C8	1.5261 (19)	C13—H13	0.9300
C2—C3	1.5416 (18)	C14—H14A	0.9600
C2—H2	0.9800	C14—H14B	0.9600
C3—C4	1.521 (2)	C14—H14C	0.9600
C3—C14	1.530 (2)	C15—H15A	0.9600
C3—C15	1.546 (2)	C15—H15B	0.9600
C4—C5	1.506 (2)	C15—H15C	0.9600
C5—C6	1.5276 (18)	C16—C21	1.379 (2)
C5—H5A	0.9700	C16—C17	1.381 (2)
C5—H5B	0.9700	C17—C18	1.383 (3)
C6—C16	1.508 (2)	C17—H17	0.9300
C6—H6	0.9800	C18—C19	1.368 (3)
C7—H7	0.9300	C18—H18	0.9300
C8—C13	1.3834 (19)	C19—C20	1.364 (3)
C8—C9	1.3856 (18)	C19—H19	0.9300
C9—C10	1.384 (2)	C20—C21	1.378 (3)
C9—H9	0.9300	C20—H20	0.9300
C10—C11	1.368 (2)	C21—H21	0.9300
			
C7—N1—C2	119.30 (11)	C9—C10—H10	119.9
C7—N1—C6	118.63 (11)	C12—C11—C10	119.29 (16)
C2—N1—C6	121.31 (10)	C12—C11—H11	120.4
N1—C2—C8	111.46 (11)	C10—C11—H11	120.4
N1—C2—C3	109.86 (10)	C11—C12—C13	120.72 (16)
C8—C2—C3	117.81 (11)	C11—C12—H12	119.6
N1—C2—H2	105.6	C13—C12—H12	119.6
C8—C2—H2	105.6	C8—C13—C12	121.03 (14)
C3—C2—H2	105.6	C8—C13—H13	119.5
C4—C3—C14	112.59 (12)	C12—C13—H13	119.5
C4—C3—C2	110.81 (12)	C3—C14—H14A	109.5
C14—C3—C2	110.75 (11)	C3—C14—H14B	109.5
C4—C3—C15	105.55 (12)	H14A—C14—H14B	109.5
C14—C3—C15	108.19 (13)	C3—C14—H14C	109.5
C2—C3—C15	108.72 (11)	H14A—C14—H14C	109.5
O2—C4—C5	121.24 (14)	H14B—C14—H14C	109.5
O2—C4—C3	122.13 (15)	C3—C15—H15A	109.5
C5—C4—C3	116.61 (11)	C3—C15—H15B	109.5
C4—C5—C6	115.08 (12)	H15A—C15—H15B	109.5
C4—C5—H5A	108.5	C3—C15—H15C	109.5
C6—C5—H5A	108.5	H15A—C15—H15C	109.5
C4—C5—H5B	108.5	H15B—C15—H15C	109.5
C6—C5—H5B	108.5	C21—C16—C17	118.60 (16)
H5A—C5—H5B	107.5	C21—C16—C6	121.60 (13)
N1—C6—C16	111.54 (11)	C17—C16—C6	119.79 (14)
N1—C6—C5	110.17 (11)	C16—C17—C18	120.33 (18)
C16—C6—C5	111.52 (12)	C16—C17—H17	119.8
N1—C6—H6	107.8	C18—C17—H17	119.8
C16—C6—H6	107.8	C19—C18—C17	120.22 (18)
C5—C6—H6	107.8	C19—C18—H18	119.9
O1—C7—N1	126.23 (14)	C17—C18—H18	119.9
O1—C7—H7	116.9	C20—C19—C18	119.9 (2)
N1—C7—H7	116.9	C20—C19—H19	120.0
C13—C8—C9	117.32 (13)	C18—C19—H19	120.0
C13—C8—C2	125.71 (12)	C19—C20—C21	120.2 (2)
C9—C8—C2	116.97 (12)	C19—C20—H20	119.9
C10—C9—C8	121.34 (14)	C21—C20—H20	119.9
C10—C9—H9	119.3	C20—C21—C16	120.67 (17)
C8—C9—H9	119.3	C20—C21—H21	119.7
C11—C10—C9	120.28 (14)	C16—C21—H21	119.7
C11—C10—H10	119.9		
			
C7—N1—C2—C8	96.72 (14)	C6—N1—C7—O1	−176.66 (14)
C6—N1—C2—C8	−93.42 (13)	N1—C2—C8—C13	100.41 (16)
C7—N1—C2—C3	−130.81 (13)	C3—C2—C8—C13	−27.9 (2)
C6—N1—C2—C3	39.05 (16)	N1—C2—C8—C9	−80.34 (14)
N1—C2—C3—C4	−55.83 (14)	C3—C2—C8—C9	151.32 (13)
C8—C2—C3—C4	73.26 (15)	C13—C8—C9—C10	−1.0 (2)
N1—C2—C3—C14	178.49 (12)	C2—C8—C9—C10	179.73 (13)
C8—C2—C3—C14	−52.43 (17)	C8—C9—C10—C11	0.5 (2)
N1—C2—C3—C15	59.74 (15)	C9—C10—C11—C12	0.5 (3)
C8—C2—C3—C15	−171.17 (12)	C10—C11—C12—C13	−0.9 (3)
C14—C3—C4—O2	−34.5 (2)	C9—C8—C13—C12	0.5 (2)
C2—C3—C4—O2	−159.11 (14)	C2—C8—C13—C12	179.78 (15)
C15—C3—C4—O2	83.37 (17)	C11—C12—C13—C8	0.4 (3)
C14—C3—C4—C5	147.10 (14)	N1—C6—C16—C21	−47.78 (18)
C2—C3—C4—C5	22.44 (17)	C5—C6—C16—C21	75.87 (17)
C15—C3—C4—C5	−95.08 (15)	N1—C6—C16—C17	132.58 (13)
O2—C4—C5—C6	−148.57 (14)	C5—C6—C16—C17	−103.77 (15)
C3—C4—C5—C6	29.89 (18)	C21—C16—C17—C18	0.0 (2)
C7—N1—C6—C16	−53.53 (16)	C6—C16—C17—C18	179.70 (14)
C2—N1—C6—C16	136.54 (12)	C16—C17—C18—C19	0.3 (3)
C7—N1—C6—C5	−177.94 (13)	C17—C18—C19—C20	−0.4 (3)
C2—N1—C6—C5	12.13 (18)	C18—C19—C20—C21	0.2 (3)
C4—C5—C6—N1	−47.61 (17)	C19—C20—C21—C16	0.1 (3)
C4—C5—C6—C16	−172.03 (12)	C17—C16—C21—C20	−0.2 (3)
C2—N1—C7—O1	−6.5 (2)	C6—C16—C21—C20	−179.87 (16)

### Hydrogen-bond geometry (Å, º)

**Table:**

*D*—H···*A*	*D*—H	H···*A*	*D*···*A*	*D*—H···*A*
C6—H6···O1^i^	0.98	2.59	3.2951 (17)	129

Symmetry code: (i) *x*−1/2, −*y*+1/2, *z*−1/2.
